# Improving Management of Type 2 Diabetes Using Home-Based Telemonitoring: Cohort Study

**DOI:** 10.2196/24687

**Published:** 2021-06-10

**Authors:** Richard Milani, Pavan Chava, Jonathan Wilt, Jonathan Entwisle, Susan Karam, Jeffrey Burton, Lawrence Blonde

**Affiliations:** 1 Center for Health Innovation Ochsner Health New Orleans, LA United States; 2 Department of Endocrinology Ochsner Health New Orleans, LA United States; 3 Center for Outcomes and Health Science Research Ochsner Health New Orleans, LA United States

**Keywords:** diabetes, digital health, ehealth, digital medicine, connected devices

## Abstract

**Background:**

Diabetes is present in 10.5% of the US population and accounts for 14.3% of all office-based physician visits made by adults. Despite this established office-based approach, the disease and its adverse outcomes including glycemic control and clinical events tend to worsen over time. Available home technology now provides accurate, reliable data that can be transmitted directly to the electronic medical record.

**Objective:**

This study aims to evaluate the impact of a virtual, home-based diabetes management program on clinical measures of diabetes control compared to usual care.

**Methods:**

We evaluated glycemic control and other diabetes-related measures after 1 year in 763 patients with type 2 diabetes enrolled into a home-based digital medicine diabetes program and compared them to 794 patients matched for age, sex, race, BMI, hemoglobin A_1c_ (HbA_1c_), creatinine, estimated glomerular filtration rate, and insulin use in a usual care group after 1 year. Digital medicine patients completed questionnaires online, received medication management and lifestyle recommendations from a clinical pharmacist or advanced practice provider and a health coach, and were asked to submit blood glucose readings using a commercially available Bluetooth-enabled glucose meter that transmitted data directly to the electronic medical record.

**Results:**

After 1 year, usual care patients demonstrated no significant changes in HbA_1c_ (mean 7.3, SE 1.7 to mean 7.3, SE 1.6; *P*=.41) or changes in the proportion of patients with HbA_1c_≥9.0 (n=117, 15% to n=113, 14%; *P*=.51). Digital medicine patients demonstrated improvements in HbA_1c_ (mean 7.3, SE 1.5 to mean 6.9, SE 1.2; *P*<.001) and significant changes in the proportion of patients with HbA_1c_≥9.0 (n=107, 14% to n=49, 6%; *P*<.001), diabetes distress (n=198, 26% to n=122, 16%; *P*<.001), and hypoglycemic episodes (n=313, 41.1% to n=91, 11.9%; *P*<.001).

**Conclusions:**

A digital diabetes program is associated with significant improvement in glycemic control and other diabetes measures. The use of a virtual health intervention using connected devices was widely accepted across a broad range of ethnic diversity, ages, and levels of health literacy.

## Introduction

The confluence of population trends, poor health outcomes, and rising costs of care make diabetes management a high global priority for health care [1]. In 2019, nearly half a billion people (9.3% of adults aged 20-79 years) were living with diabetes worldwide, representing a 62% increase from 10 years ago [2]. According to the US Centers for Disease Control and Prevention and the 2016 National Ambulatory Medical Care Survey, diabetes is the seventh leading cause of death, present in 10.5% of the US population, and accounts for 14.3% of all office-based physician visits made by adults [3,4]. Diabetes ranks the highest among all disease categories in health care spending, with expenditures per capita approximately 2.3 times higher than people without diabetes [3,5,6]. Moreover, 40% of the cost of diabetes is associated with the cohort whose hemoglobin A_1c_ (HbA_1c_) values are 9.0% or greater [7].

Although progress has been made to improve risk factors for microvascular and macrovascular disease in diabetes, approximately half of individuals with diabetes do not meet individualized targets for HbA_1c_, and less than 15% meet all three targets of glycemic control, blood pressure, and low-density lipoprotein (LDL) cholesterol [8-10]. Moreover, outcomes such as emergency department visits, lower extremity amputations, hospitalizations for hyperglycemic crisis, and deaths due to diabetes have worsened over the past decade [10,11].

Several factors account for these poor outcomes, including the use of suboptimal numbers or doses of medications, therapeutic inertia, lack of patient engagement, and limited resources and time to educate and provide lifestyle recommendations [12,13]. Although many types of interventions have been tested, recent reviews conclude that what is needed is a reorganization of clinical practice using care teams that empower nonphysician clinicians to adjust diabetes therapy from algorithms developed in collaboration with other team members [14-16].

Home blood glucose monitoring with direct transmission of data to the medical record addresses several limitations of traditional office-based care, including a larger sample of biologic data and an ability to take more timely action including therapy modifications [17-19]. Current technology including Bluetooth-enabled digital glucometers is accurate and easy to use, and automatically transmitted home-based glucose measurements have the capacity to better identify hypoglycemic events.

We sought to evaluate the effectiveness of a remote, home-based telemonitoring program in a clinical setting using commercially available technologies on glycemic control in adults with type 2 diabetes (T2D).

## Methods

As part of a quality improvement initiative, adult patients with the diagnosis of T2D at the Ochsner Health System (a large integrated delivery network based in New Orleans, LA) were offered enrollment into a digital diabetes program by their physician during an office encounter or through an offer letter. Patients were required to possess a smartphone, purchase a Bluetooth connected glucose meter (iHealth Smart) that had direct access to the electronic medical record (EMR; Epic Systems), and have an active account in the patient portal (MyChart). If patients did not have an active patient portal account, they were given the opportunity and online assistance to sign up for one. Program details, questionnaires, and electronic consent to participate took place online through MyChart. Questionnaires assessed factors related to diabetes and chronic disease management, including diet, physical activity, depression, medication adherence, patient activation, health literacy, and social circumstances (eg, medication affordability and number of people living in home). Diabetes-related emotional distress was assessed using the Diabetes Distress Scale [20,21]. Health literacy was measured using the single item literacy screener [22]. Additional clinical data was obtained from the EMR, including serum glucose, HbA_1c_, lipid levels, creatinine, estimated glomerular filtration rate (eGFR), thyroid function tests, urine protein, and BMI as well as completion of a retinal examination. This data was used to create a patient phenotype that assisted in the design of the intervention process. As an example, a poorly controlled individual with low health literacy and financial stress would be differentiated from an individual with high patient activation and reduced physical activity. Digital glucose meters were obtained, and initial training and setup was provided at the Ochsner O Bar, a patient-facing service that provides information, training, and tech support for patients interested in apps, wearables, and connected home devices [17]. All blood glucoses obtained via the patient’s home glucometer were automatically transmitted via Bluetooth to the MyChart phone app and thus were available to the management team within the medical record. Hypoglycemia was defined as a blood glucose measurement less than 70 mg/dl, level 1 hypoglycemia as 54 to 70 mg/dl, level 2 as 40 to 54 mg/dl, and level 3 as less than 40 mg/dl.

A second control group of patients who met all eligibility criteria but whose physician was either not participating in the program or did not choose to enroll patients were followed. Of these, 794 patients were propensity matched to the digital medicine group according to age, sex, race, BMI, HbA_1c_, creatinine, eGFR, and insulin use, and were followed as a usual care group over time.

Doctoral pharmacists, advanced practice providers (APPs) including nurse practitioners and physician assistants, and health coaches participated in the intervention that included education, lifestyle recommendations (medical nutrition therapy and appropriately prescribed physical activity), and medication management as per diabetes guidelines [16,23]. Each health coach had a background in a lifestyle intervention field (eg, nutrition and exercise specialist) or in public health.

Each doctoral pharmacist, APP, and health coach received training in diabetes management and education as well as use of the custom tools within the EMR created to facilitate optimal management [17,19]. Doctoral pharmacists, APPs, and health coaches were also educated regarding the importance of patient activation and methods used to enhance engagement and lifestyle change [24-26].

Pharmacists and APPs contacted patients by phone and discussed screening results and treatment options for achieving glycemic control. Patients were encouraged to actively participate in their diabetes management and collaborated with the doctoral pharmacist or APP to cocreate the treatment plan by choosing among various lifestyle and medication recommendations [27]. Current American Diabetes Association guidelines were used by the pharmacy team for medication management [28]. Patients were also directed to a dedicated diabetes education website that offered further educational and lifestyle materials including custom videos and downloadable handouts.

Patients with medication affordability issues were, as much as possible, switched to generics or less expensive combination agents and, when appropriate and feasible, enrolled in medication assistance programs. Those with medication adherence issues were provided educational materials, pill reminder apps and resources, and a simplified medication regimen when possible.

Patients received monthly reports by mail and electronically detailing their progress, including HbA_1c_ and upcoming health maintenance metrics as well as lifestyle tips based on their screening phenotype. Physicians also received monthly reports on their patient’s progress including blood glucose control and health maintenance metrics. Incoming glucose data was analyzed via internally developed algorithms as to its validity and directional change, and alerts were established to highlight which patients needed what intervention and when. Individualized targets for HbA_1c_ were created based on current guideline recommendations [16].

The primary outcome was the proportion of patients achieving their glycemic control. Secondary outcomes included incidence of hypoglycemia and achievement of health maintenance measures (HbA_1c_ measurements, retinal imaging for retinopathy, and screening for diabetic kidney disease).

To assess association between the digital medicine program for diabetes and patient outcomes, general linear models were used [29]. Distributions of outcomes were assessed, and all were approximately normally distributed. For each outcome, an unadjusted model containing only a main effect for digital medicine (vs usual care) and a multivariable model incorporating covariates were constructed. The multivariable model included covariates for patient age, sex, and race. Additional covariates for baseline fasting glucose and baseline eGFR were included in the model for HbA_1c_. The response variable in each model was the change in the outcome from baseline to 12 months. Results are presented as means and SEs of the changes within each group (digital medicine and usual care), differences in group means, 95% CIs, and *P* values. To evaluate changes in medications from baseline to 12 months, a logistic model for repeated measures was constructed [30]. The model included main effects for digital medicine (vs usual care) and time and the digital medicine × time interaction. No covariates were considered. The multivariate binary response indicated medication status (yes or no) at baseline and 12 months. A compound symmetric covariance structure was used to model within-patient correlation. Results are presented as odds ratios of medication (12 months vs baseline) within each group, ratios of the group odds ratios, 95% CIs, and *P* values. Because no patients in the digital medicine program were treated with alpha glucosidase inhibitors, this medication class was not included in the analysis. A significance level of .05 was used for all statistical tests. Analyses of outcomes were carried out using SAS version 9.4 for Windows (SAS Institute).

## Results

We evaluated all patients at baseline and again following 1 year. Prior to enrollment in the digital diabetes program, patients were under the care of their primary care clinician for their diabetes for an average of 5.2 years, averaging 2.8 visits per year. Of the 763 patients at baseline, 328 (43%) patients had not achieved their goal HbA_1c_ target, while 107 patients (14%) had HbA_1c_ values greater than or equal to 9.0% (range 9.0-13.9).

The baseline characteristics of the digital diabetes (n=763) and the propensity matched usual care groups (n=794) are outlined in Table 1. There were no significant differences in age, sex, BMI, HbA_1c_, creatinine, eGFR, and use of insulin. There were, however, lower levels of total and high-density lipoprotein (HDL) cholesterol as well as higher systolic and diastolic blood pressure in digital medicine patients. It is noteworthy that digital medicine patients’ age ranged from 31 to 98 years, with 28% (n=215) of enrollees 70 years or older.

**Table 1:** Baseline comparison of digital medicine (n=763) and usual care (n=794).

Metric	Digital medicine	Usual care	Standard difference	*P* value
Age (years), mean (SE)	63 (11)	63 (12)	0.031	.54
Male, n (%)	373 (49)	357 (45)	0.068	.11
Black, n (%)	299 (39)	310 (39)	0.013	.99
White, n (%)	447 (59)	468 (59)	0.002	.99
Fasting glucose (mg/dl), mean (SE)	155 (67)	151 (64)	0.065	.20
HbA_1c_^a^ (%), mean (SE)	7.3 (1.5)	7.3 (1.7)	0.020	.70
Creatinine (mg/dl), mean (SE)	1.06 (0.4)	1.03 (0.04)	0.057	.76
Estimated glomerular filtrate rate (ml/min), mean (SE)	58.5 (9.5)	58.6 (9.1)	0.012	.44
Total cholesterol (mg/dl), mean (SE)	161 (38)	165 (44)	0.107	.04
HDL^b^ cholesterol (mg/dl), mean (SE)	45 (12)	46 (13)	0.109	.04
LDL^c^ cholesterol (mg/dl), mean (SE)	92 (49)	95 (50)	0.050	.34
Triglycerides (mg/dl), mean (SE)	146 (95)	149 (150)	0.021	.69
BMI (kg/m^2^), mean (SE)	34.7 (7.3)	35.0 (8.3)	0.034	.51
Systolic blood pressure (mmHg), mean (SE)	132 (13)	130 (14)	0.101	.05
Diastolic blood pressure (mmHg), mean (SE)	78 (8)	76 (8)	0.196	<.001

^a^HbA_1c_: hemoglobin A_1c_.

^b^HDL: high-density lipoprotein.

^c^LDL: low-density lipoprotein.

Additional characteristics were uniquely obtained in digital medicine patients. At entry, 168 of the 763 (22%) patients exhibited symptoms of depression, 160 (21%) patients described financial difficulty in paying for medication, and 122 (16%) patients had low levels of health literacy. Basic technology skills were assessed at entry, of which 69 (9%) patients scored as deficient [31,32]. Moderate to high levels of diabetes distress was reported in 198 (26%) patients at entry, which improved to 122 (16%) patients at 1 year (*P*<.001). Finally, a net promotor score (likelihood to recommend the program to a friend or colleague) survey was collected yielding 385 responses (50% response rate), generating a score of 83 (range –100 to 100).

Tables 2 and 3 describe the impact of digital medicine and usual care on key metrics. Usual care patients demonstrated no improvements in HbA_1c_, fasting glucose, or blood pressure but did show improvements in total cholesterol and BMI.

**Table 2:** Impact of digital medicine management at 12 months.

Metric	Baseline, mean (SE)	12 months, mean (SE)	Change (%)	*P* value
Fasting glucose (mg/dl)	155 (67)	137 (47)	–12	<.001
HbA_1c_^a^ (%)	7.3 (1.5)	6.9 (1.2)	–7	<.001
Total cholesterol (mg/dl)	161 (38)	154 (37)	–4	<.001
HDL^b^ cholesterol (mg/dl)	45 (12)	46 (13)	5	<.001
LDL^c^ cholesterol (mg/dl)	92 (49)	86 (52)	–6	.02
Triglycerides (mg/dl)	146 (95)	140 (87)	–7	.04
BMI (kg/m^2^)	34.7 (7.3)	34.4 (7.1)	–1	<.001
Systolic blood pressure (mmHg)	132 (13)	131 (10)	–1	.31
Diastolic blood pressure (mmHg)	78 (8)	77 (7)	–1	<.001

^a^HbA_1c_: hemoglobin A_1c_.

^b^HDL: high-density lipoprotein.

^c^LDL: low-density lipoprotein.

**Table 3:** Impact of usual care at 12 months.

Metric	Baseline, mean (SE)	12-months, mean (SE)	Change (%)	*P* value
Fasting glucose (mg/dl)	151 (64)	151 (66)	0	.61
HbA_1c_^a^ (%)	7.3 (1.7)	7.3 (1.6)	0	.41
Total cholesterol (mg/dl)	165 (44)	160 (40)	–3	.02
HDL^b^ cholesterol (mg/dl)	43 (13)	46 (14)	7	.25
LDL^c^ cholesterol (mg/dl)	95 (50)	96 (64)	1	.33
Triglycerides (mg/dl)	149 (150)	152 (139)	2	.46
BMI (kg/m^2^)	35.0 (8.3)	34.6 (8.2)	–1	<.001
Systolic blood pressure (mmHg)	130 (14)	131 (12)	0.8	.21
Diastolic blood pressure (mmHg)	76 (8)	76 (7)	0	.68

^a^HbA_1c_: hemoglobin A_1c_.

^b^HDL: high-density lipoprotein.

^c^LDL: low-density lipoprotein.

Digital medicine patients achieved significant improvements in HbA_1c_, lipids, BMI, and diastolic blood pressure, which took place without changes in lipid or antihypertensive therapy. Digital medicine management yielded a 57% reduction in the percent of patients whose HbA_1c_≥9.0% (14% to 6%; *P*<.001), whereas no statistically significant change was observed in usual care patients (15% to 14%; *P*=.51; Table 4).

Digital medicine patients were significantly more likely to complete annual health maintenance measures (Table 5) than those in usual care.

Tables 6 and 7 describe medication use and changes in both digital diabetes and usual care groups.

Medication changes were similar in both groups except for a greater reduction in sulfonylurea use in the digital medicine group (Table 8).

**Table 4:** Proportion of patients in digital medicine (n=763) and usual care (n=794) whose hemoglobin A_1c_≥9.0%.

Group	Baseline	12 months	Change (%)	*P* value
Digital medicine, n (%)	107 (14)	49 (6)	–54	<.001
Usual care, n (%)	117 (15)	113 (14)	–3	.51

**Table 5:** Proportion of patients completing annual health maintenance in the digital medicine (n=763) and usual care (n=794) groups.

Annual health maintenance metric	Digital medicine	Usual care	*P* value
HbA_1c_^a^, n (%)	684 (90)	584 (74)	<.001
Retinopathy exam, n (%)	649 (85)	554 (70)	<.001
Urine protein, n (%)	746 (98)	751 (95)	.001

^a^HbA_1c_: hemoglobin A_1c_.

**Table 6:** Changes in diabetes medications over 12 months of digital medicine management.

Medication class	Baseline	12 months	Change (%)	*P* value
Insulin (%)	27	24	–13	.10
Biguanide (%)	70	56	–20	<.001
Sulfonylureas (%)	27	15	–43	<.001
GLP-1^a^ agonists (%)	25	33	30	.001
DPP-4^b^ inhibitors (%)	14	8	–43	<.001
SGLT-2^c^ inhibitors (%)	11	18	67	<.001
Alpha glucosidase inhibitors (%)	0	0	0	>.99
Thiazolidinedione (%)	4	3	–18	.48

^a^GLP-1: glucagon-like peptide 1.

^b^DPP-4: dipeptidyl peptidase 4.

^c^SGLT-2: sodium-glucose cotransporter 2.

**Table 7:** Changes in diabetes medications over 12 months in usual care.

Medication class	Baseline	12 months	Change (%)	*P* value
Insulin (%)	25	23	–12	.41
Biguanide (%)	64	49	–23	<.001
Sulfonylureas (%)	23	16	–31	<.001
GLP-1^a^ agonists (%)	16	20	27	.03
DPP-4^b^ inhibitors (%)	13	8	–39	.001
SGLT-2^c^ inhibitors (%)	9	12	38	.03
Alpha glucosidase inhibitors (%)	0.3	0.1	–50	.56
Thiazolidinedione (%)	4	3	–30	.17

^a^GLP-1: glucagon-like peptide 1.

^b^DPP-4: dipeptidyl peptidase 4.

^c^SGLT-2: sodium-glucose cotransporter 2.

**Table 8:** ORs of medication use at 12 months by group.

Outcome	Digital medicine, OR^a,b^ (95% CI)	Usual care, OR (95% CI)	Ratio^c^ (95% CI)	*P* value
Insulin	0.82 (0.74-0.92)	0.91 (0.81-1.01)	0.91 (0.78-1.06)	.22
Biguanide	0.54 (0.47-0.63)	0.54 (0.47-0.62)	1.00 (0.82-1.22)	>.99
Sulfonylureas	0.49 (0.42-0.57)	0.63 (0.54-0.74)	0.78 (0.62-0.97)	.02
GLP-1^d^ agonists	1.45 (1.26-1.66)	1.34 (1.14-1.57)	1.08 (0.87-1.34)	.47
DPP-4^e^ inhibitors	0.53 (0.42-0.66)	0.58 (0.46-0.72)	0.92 (0.67-1.26)	.60
SGLT-2^f^ inhibitors	1.83 (1.51-2.21)	1.43 (1.16-1.78)	1.27 (0.95-1.70)	.10
Alpha glucosidase inhibitors	—^g^	—	—	—
Thiazolidinedione	0.82 (0.61-1.10)	0.69 (0.52-0.92)	1.18 (0.78-1.78)	.44

^a^OR: odds ratio.

^b^ORs for groups are odds of using the medication at 12 months divided by odds of using the medication at baseline.

^c^Ratio (95% CI) is the OR for the digital medicine divided by the OR for the usual care group along with the 95% CI.

^d^GLP-1: glucagon-like peptide 1.

^e^DPP-4: dipeptidyl peptidase 4.

^f^SGLT-2: sodium-glucose cotransporter 2.

^g^Not available.

Baseline and 1-year hypoglycemic episodes were recorded in digital medicine patients (Table 9). A total of 313 (41%) patients experienced at least 1 hypoglycemic event per month, averaging 0.84 episodes per month per patient enrolled, of which 6% were level 3 hypoglycemic events. At 1 year, there was a 74% reduction (*P*<.001) in total hypoglycemic episodes with a commensurate 82% reduction in frequency to 0.15 episodes per patient per month (*P*<.001).

The average change in these metrics per patient are described in Table 10, and Table 11 adjusts these changes based on patient age, sex, and race. Following adjustment, there were statistically greater improvements observed in fasting glucose, HbA_1c_, HDL and LDL cholesterol, and diastolic blood pressure in the digital medicine managed patients compared to usual care.

**Table 9:** Changes in hypoglycemic events over 1 year in digital medicine patients (n=763).

Monthly hypoglycemia			Baseline		12 months	Change (%)	*P* value
**Patients with any hypoglycemic episode, n (%)**			314 (41.1)		91 (11.9)	–74	<.001
	Patients with level 1 episodes	175 (22.9)		63 (8.3)		–64	<.001
	Patients with level 2 episodes	93 (12.2)		16 (2.1)		–83	<.001
	Patients with level 3 episodes	46 (6.0)		11 (1.5)		–75	<.001
Frequency of hypoglycemic episodes per month, mean			0.84		0.15	–82	<.001

**Table 10:** Unadjusted mean within-patient change in outcomes by group.

Outcome	Digital medicine, mean (SE)	Usual care, mean (SE)	Difference (95% CI)	*P* value
Fasting glucose	–17.7 (2.7)	1.3 (2.5)	–19.0 (–26.2 to –11.7)	<.001
HbA_1c_^a^	–0.40 (0.05)	0.04 (0.05)	–0.44 (–0.58 to –0.30)	<.001
Total cholesterol	–5.9 (1.2)	–3.7 (1.6)	–2.2 (–6.1 to 1.7)	.27
HDL^b^ cholesterol	1.6 (0.3)	0.4 (0.3)	1.2 (0.4 to 2.1)	.003
LDL^c^ cholesterol	–5.1 (2.2)	2.7 (2.8)	–7.8 (–14.8 to –0.8)	.03
Triglycerides	–6.5 (3.2)	4.9 (6.6)	–11.4 (–25.8 to 3.0)	.12
BMI	–0.25 (0.06)	–0.30 (0.08)	0.05 (–0.14 to 0.24)	.62
Systolic BP^d^	–0.38 (0.38)	0.52 (0.41)	–0.90 (–2.00 to 0.20)	.11
Diastolic BP	–0.87 (0.22)	–0.09 (0.23)	–0.77 (–1.39 to –0.15)	.01

^a^HbA_1c_: hemoglobin A_1c_.

^b^HDL: high-density lipoprotein.

^c^LDL: low-density lipoprotein.

^d^BP: blood pressure.

**Table 11:** Covariate-adjusted mean within-patient change in outcomes by group.

Outcome	Digital medicine^a^, mean (SE)	Usual care, mean (SE)	Difference (95% CI)	*P* value
Fasting glucose	–17.4 (2.7)	1.1 (2.5)	–18.5 (–25.7 to –11.2)	<.001
HbA_1c_^b,c^	–0.38 (0.05)	0.03 (0.05)	–0.41 (–0.54 to –0.27)	<.001
Total cholesterol	–5.8 (1.2)	–3.8 (1.6)	–2.0 (–6.0 to 1.9)	.31
HDL^d^ cholesterol	1.6 (0.3)	0.3 (0.3)	1.3 (0.5 to 2.1)	.002
LDL^e^ cholesterol	–4.9 (2.2)	2.5 (2.7)	–7.5 (–14.4 to –0.5)	.04
Triglycerides	–6.7 (3.2)	5.1 (6.6)	–11.8 (–26.4 to 2.7)	.11
BMI	–0.25 (0.06)	–0.30 (0.08)	0.05 (–0.14 to 0.25)	.59
Systolic BP^f^	–0.40 (0.38)	0.53 (0.41)	–0.93 (–2.03 to 0.17)	.10
Diastolic BP	–0.86 (0.22)	–0.10 (0.23)	–0.76 (–1.38 to –0.14)	.02

^a^All models incorporate covariates for patient age, sex, and race.

^b^HbA_1c_: hemoglobin A_1c_.

^c^Model for HbA_1c_ incorporated additional covariates for baseline fasting glucose and baseline estimated glomerular filtration rate.

^d^HDL: high-density lipoprotein.

^e^LDL: low-density lipoprotein.

^f^BP: blood pressure.

## Discussion

There are several important findings from this investigation. First, the digital health monitoring and intervention program significantly improved HbA_1c_ levels, attainment of goal HbA_1c_, lipid levels, diabetes distress, and annual health maintenance adherence compared to usual care. Second, hypoglycemia is frequently present and is often unrecognized in individuals with diabetes managed in clinical practice; the severity and frequency of hypoglycemic events can be markedly reduced with timely knowledge and intervention through real-time capture of home self-monitoring of blood glucose [33,34]. Finally, a successful digital managed diabetes program need not be limited to a younger, more technology-advanced population, and can fully embrace a wide range of racial diversity, age, and health literacy levels with high levels of patient satisfaction.

The standard management of diabetes in clinical practice has significant system and process limitations including limited episodic interactions with patients, access to a small fraction of glucose data, a reduced ability to evaluate patient comprehension and needs, and a brief amount of time to educate and reinforce essential health-promoting behaviors [12,33-36]. Physicians report that they are less satisfied providing care to people with chronic conditions such as diabetes than to patients in general, which may result from difficulty in care coordination, inadequate clinical training, and poor reimbursement for the time necessary [37,38]. As a result, over the past decade, the United States has experienced worsening of clinical outcomes in diabetes, including an increase in emergency department visits, lower extremity amputations, hospitalizations for hyperglycemic crisis, and deaths due to diabetes [10,11].

We have demonstrated that redesigning care delivery using a digital health program can substantially improve multiple metrics important in caring for patients with T2D, namely, achievement of HbA_1c_ targets, reduction of hypoglycemia, reducing diabetes distress, and completing necessary health maintenance. These improvements are in contrast to outcomes that often occur when people with diabetes are seen only 2 to 3 times a year, resulting in persistence of hyperglycemia or hypoglycemia over prolonged periods of time [33,34]. This was evidenced in the usual care group where the percentage of patients with the highest levels of HbA_1c_ remained unchanged after 1 year. Moreover, after an average of 5 years under the care of their primary care physician, 41% (n=314) of patients were discovered to have hypoglycemic events each month, of which 6% (n=46) were level 3. Finally, the digital intervention was provided to a diabetes population with a broad range of ages (range 31-98 years), races, and health literacy, and it overcame many potential barriers to care, including access.

There are several factors worth describing in our care delivery redesign. First, the clinical team of doctoral pharmacists and APPs prescribed medications based on current guideline recommendations, which contrasts the high rate of care variation observed in routine practice [39-42]. Second, adjustments to guideline-recommended medication used glucose values received directly from home, providing the clinical team with just-in-time actionable data. This improved the ability to recognize and reduce both hypoglycemia and hyperglycemia. Third, each patient benefited from a dedicated health coach proficient in lifestyle intervention and skills to improve health literacy; patient activation; and, when possible, financial strain related to the cost of medications [12,31]. Fourth, patients received monthly reports outlining their status regarding diabetes control and additional tips and reminders needed for optimal outcomes. Finally, most interactions took place during openings in the patient’s schedule rather than openings in the care team’s schedule, making receiving care highly convenient and desirable [18].

It is noteworthy that costs of care were not assessed, and future studies will be needed to determine the financial impact of this digital intervention.

There are, however, several limitations to this study. First, this was a single-center study with a 1-year follow-up occurring in an integrated health care delivery system and involved a relatively modest number of individuals whose average glycemic, blood pressure, and lipid control were not markedly abnormal. Despite the advantages of Bluetooth-enabled glucose meters, it is likely that the use of continuous glucose monitoring (CGM) would have identified a greater number of hypoglycemic events. With recent improvements in CGM performance and availability, it is likely this technology will be offered to appropriate participants in the future. Second, patients were not prospectively randomized into intervention and usual care groups. Finally, only patients who possessed a smartphone were eligible to enroll, which raises issues about education, socioeconomic, and motivational biases. However, the mean age of our population was 63 years, and on screening, 9% (n=69) lacked common technology skills, suggesting that our cohort was not biased toward a younger, more technically competent population.

In summary, a team-based digital health program that incorporates a higher frequency of real-time glucose data and touchpoints with the clinical team is an effective modality for delivering diabetes management, outperforming traditional office-based care. Although the bulk of our results were obtained prior to the COVID-19 pandemic, this mode of care provides unique value in this setting.

## Abbreviations

APP: advanced practice provider
CGM: continuous glucose monitoring
eGFR: estimated glomerular filtration rate
EMR: electronic medical record
HbA1c: hemoglobin A1c
HDL: high-density lipoprotein
LDL: low-density lipoprotein
T2D: type 2 diabetes
